# From urban ecology to urban enquiry: How to build cumulative and context-sensitive understandings

**DOI:** 10.1007/s13280-023-01959-5

**Published:** 2024-04-20

**Authors:** Erik Andersson, Timon McPhearson, Steward T. A. Pickett

**Affiliations:** 1 Ecosystems and Environment Research Programme, University of Helsinki, Viikinkaari 1, P.O. Box 65, 00014 Helsinki, Finland; 2 Stockholm Resilience Centre, Stockholm University, Albanovägen 28, 10691 Stockholm, Sweden; 3 Research Unit for Environmental Sciences and Management, North-West University, Private Bag X6001, Potchefstroom, 2520 South Africa; 4 Urban Systems Lab, The New School, 79 Fifth Avenue, 16th Fl., New York, NY 10003 USA; 5 Cary Institute of Ecosystem Studies, Box AB, Millbrook, NY 12545 USA

**Keywords:** Commensurability, Interdisciplinarity, Mixed methods, Multiple evidence, Social–ecological–technological systems, Systems thinking

## Abstract

This paper positions urban ecology as increasingly conversant with multiple perspectives and methods for understanding the functions and qualities of diverse cities and urban situations. Despite progress in the field, we need clear pathways for positioning, connecting and synthesising specific knowledge and to make it speak to more systemic questions about cities and the life within them. These pathways need to be able to make use of diverse sources of information to better account for the diverse relations between people, other species and the ecological, social, cultural, economic, technical and increasingly digital structures that they are embedded in. Grounded in a description of the systemic knowledge needed, we propose five complementary and often connected approaches for building cumulative systemic understandings, and a framework for connecting and combining different methods and evidence. The approaches and the framework help position urban ecology and other fields of study as entry points to further advance interdisciplinary synthesis and open up new fields of research.

## Introduction

The shift in urban ecology from being interested in ecosystems embedded in urban landscapes (like e.g. Blair 1996; Marzluff 2005) to urban systems as ecosystems (Pickett et al. 1997b; Grimm et al. 2008) meant a fundamental change in the information needed to answer a wider scope of questions. As an increasingly transdisciplinary field, urban ecology, thus needed to become conversant in and compatible with discussions about justice, identity, wellbeing, urban resilience and sustainability (e.g. Grimm et al. 2000; McHale et al. 2015; McPhearson et al. 2016a, b; Schell et al. 2020; Shackleton and Cocks 2020). Overlapping interests between disciplines and epistemological traditions show that there are different ways of answering questions, each with their limitations and opportunities (Rademacher et al. 2023). Finding the best fit with a given situation, understanding the type of answer you get, and being able to compare and combine with knowledge derived through other means are an essential step towards advancing an urban ecology that more explicitly addresses the critical importance of social and technological dimensions of urban systems (McPhearson et al. 2016a, b). Expectations on richer contextualisation and deeper discussions about, for example, the justice and wellbeing implications of the distribution of urban green infrastructure reinforce the need for interdisciplinary approaches and integrated methodological designs (e.g. Andersson et al. 2021a, b, c). Challenging as it may be, we see this increasing convergence and overlap between expanding fields of urban study as a promising foundation for connecting and cross-fertilising multiple ways of building more comprehensive, systemic understandings.

Global data coverage as well as opportunities and capacity to explore in depth the nature and qualities of urban systems are unevenly distributed, with issues of data gaps or insufficient theory, resources, and capacity constraining how we can understand processes and patterns of cities and urbanised areas (Jacobs et al. 2016; Creutzig et al. 2019). Ideally, research should be able to account both for a local case with all its complexities and situatedness *and* contribute to the general understanding of the ‘urban’ and its commonalities (an old and persistent challenge, see e.g. Merton 1968). At the same time, the urgent need to address current environmental and sustainability challenges calls for learning across urban contexts and translating findings and tentative solutions to other cities (McPhearson et al. 2021a, b). This will mean using different data and drawing on different competences and resources to answer similar questions in different contexts, while finding appropriate methods to derive the best possible answers from the data we have or can realistically acquire.

The argument in this perspective article is that a systems approach does not always mean starting with the wider, systemic picture or trying to include all aspects of a system at once. Systemic understandings can also be built incrementally by contextualising and expanding the scope of narrow, targeted research questions (see e.g. Vayda 1984), gradually addressing additional questions or adding new layers of interpretation and association. We focus on the academic understanding, other papers in this Special Feature (Pickett et al. 2024; Frantzeskaki et al. 2024) expand on the transdisciplinary and action-oriented side of the discussion.

Systems approaches have been challenged as to their ability to predict causality (e.g. Levin 1998) and yet accused of being deterministic and simplifying local complexity (e.g. Parnell and Oldfield 2014). They have also been promoted for their ability to connect and relate different features and point to critical dynamics (e.g. McPhearson et al. 2022). Work on expanding and adding to existing cases and research perspectives can lead towards consonance by building thematic, geographic, methodological, or practical overlaps and connections. Here, the systems approach is rather the provision of a set of ‘coordinates’ for positioning and starting to relate different perspectives and insights. Urban systems and the relation between people from different socio-cultural contexts and differently framed and understood urban natures have been conceptualised in many different ways such as the metacity (McGrath and Pickett 2011), cities as social–ecological systems (e.g. Berkes and Folke 1998; Levin et al. 2013; Andersson et al. 2014) world cities (Friedmann 1986) and ordinary cities (Robinson 2006), socio-technical systems (e.g. Geels 2004), and urban metabolism (e.g. Kennedy et al. 2011), each attempting to provide guidance for how to reveal urban patterns and capture essential urban processes and dynamics. Such models and conceptual frameworks have been reviewed elsewhere (e.g. Zhou et al. 2021).

Instead, we argue that rapid global development and concomitant global change calls for pragmatism, and for making the most we can of the evidence we have, heterogeneous as it may be. This paper positions urban ecology as a thread in an increasingly multi-faceted and holistic approach to understanding the functions and qualities of the heterogeneous population centres grouped under the label ‘cities’. We need clear pathways for embedding and connecting ecology to the social, cultural, economic, technical, and increasingly digital structures of diverse cities, and using diverse sources of information to better account for the diverse relations between people, nature and urban infrastructures. Drawing on a wide range of urban scholarship, we start with outlining a conceptual basis and then move on to a set of complementary methodological approaches we believe can help us do more with the evidence we have.

### Conceptually rich and methodologically challenging

Urban ecology may at first seem to be a biological science, given that the discipline of ecology emerged by combining laboratory physiology of animals and plants, field distributions and limits of organisms, the flow of energy and nutrients in the field, and changes in adaptation or organismal communities through time. Urban ecology’s early roots focused on such questions and approaches in those specific habitats within cities where the methods were applicable. This has been called “ecology in the city” (Pickett et al. 1997a). But urban ecologists have recognised for a long time that they need to draw upon details of social structure, institutional arrangements, political and economic interactions to understand what they were learning about plants, animals, and biogeochemistry in and around cities. Simple human demographic parameters measured adjacent to forests, streams and wetlands of ecological interest were inadequate. The term “ecology of cities” came to represent the expanded ecological view of the field (Pickett et al. 1997a; Grimm et al. 2000; Groffman et al. 2017).

With this as the background, we put the equitable access to and sustainable use of ecosystem-based wellbeing benefits, in recognition of the intrinsic values and rights of the biosphere itself, as the thematic backbone of our envisioned systems understanding. This conceptualisation (known by many as the ecosystem service cascade, sensu Haines-Young and Potschin 2010) positions studies along a continuum from ecosystems and ecological processes (with or without human involvement) to people and the embodied experiences and sometimes imaginary ‘nature’ that influence our relationship with our surroundings. In between are all the mediating factors influencing the realisation of ecosystem service potential as different benefits (Haines-Young and Potschin 2010; Andersson et al. 2019; Keeler et al. 2019). Our systems approach thus positions ‘ecological’ dynamics and their social–ecological outcomes as something that partly emerges from, and must be connected to, human everyday life. For example, human life in cities and urban lifestyles have become increasingly distant from active, intentional creation and realisation of different ecosystem-based wellbeing benefits and, thus, have lost nuanced and richer experiences of nature (sensu Miller 2005; Soga and Gaston 2016).

Since the shift towards ‘ecology of cities’, there has been a further turn towards relational, digital and globally plural ways of understanding the city and nature’s role in it (Pickett et al. 2022), all pointing to critical—and understudied or yet unconnected—aspects we need to include as we move urban ecology forward.

#### The turn: Richer and more complex

While the conceptualisation of urban ecology as embedded and interdependent has been in active use across disciplines—including human ecology, social–ecological systems studies, environmental psychology, ecological urbanism, and ecological engineering—for quite some time, recent years have seen the emergence of more relational perspectives (e.g. Poe et al. 2014; West et al. 2020) and a merging of social–ecological thinking and socio-technical studies (Wolfram et al. 2016; McPhearson et al. 2021a, b). Ecological properties are often processed and transmuted through active human mediation, from hands-on management to sense- and place-making and value attribution, and thus, become woven into urban life. Recognition of ecosystem services as emergent qualities of human–nature relations (e.gSpirn 1984; Haraway 2003; Ernstson 2013; Palomo et al. 2016) connects ecology to planning and governance, and the human involvement in the production and distribution of ecosystem service benefits also fit well with a discussion about environmental justice (Langemeyer and Connolly 2020). Nature is now positioned as a provider of benefits to people (Pascual et al. 2017) and a basis for tentative solutions to various urban challenges (IUCN 2020; Palomo et al. 2021; Dodman et al. 2022; Kabisch et al. 2022), implicitly asking the question ‘for whom?’. For example, in New York City, examining the supply and demand for a diverse array of ecosystems services reveals stark contrasts and clear distributional injustices when comparing the high local supply and low demand for ecosystem services in neighbourhoods that are predominantly wealthy and white to the high demand and low supply in neighbourhoods that are predominantly low-income and communities of colour (Herreros‐Cantis and McPhearson 2021). In parallel, cities are also discussed as places where biodiversity must be given more recognition, voice and rights (e.g. Wolch et al. 1995; Borràs 2016; Houston et al. 2018; Cooke et al. 2020; Pineda-Pinto et al. 2022). Recognising biodiversity’s intrinsic rights, also to our cities, challenges conventional urban planning and development.

There is a general agreement that the young and old, expanding and shrinking, differently planned and organically evolved cities across the globe diverge in their characteristics (Elmqvist et al. 2013). In fact, diversity among the cities of the world is one of the most conspicuous features of the current state of urbanisation (McHale et al. 2015). Cities have different origins, sometimes being established de novo at locations favourable for defence, access to trade routes (especially rivers and harbours) or located “on top of” older agricultural settlements. In addition to a variety of origins, cities have diverse trajectories through time. A familiar trajectory in the wealthy nations of the global north is from a market town, evolving into a mercantile city, then to an industrial centre, and sometimes after that a city based on service rather than material production. In cities of more recent origin, or in regions of the Global South, there is no typical trajectory of urban change, and many such cities there have recently emerged as cities of post-colonial liberation, or of consumption, opportunity, and refuge.

In addition to these cultural, socioeconomic and historical differences, cities located in different ecoregions have different ecological baselines, and cities are often characterised by novel ecosystems merging legacies of pre-urban ecosystems with human-induced (intentionally or not) organisms and novel biophysical conditions (e.g. Hobbs et al. 2006; Ahern 2016). These emerging ecosystems are meshed with built urban infrastructures and layers of human meaning making, use and governance. Recent decades have seen increasing attention to the world of cities, challenging decontextualised and uncritical transfer of theories and best practices (in research and for urban development alike) (e.g. Parnell and Oldfield 2014). The cities with the longest history of documented studies are no longer the places where we see the most critical contemporary urban problems. Instead, they unfold in situations “where traditional authority, religious identity or informality are as central to legitimate urban narratives as the vacillations in modern urban capitalist public policy” (ibid, p. 2). While often led by a South–North perspective, the shift and its regional logic can be understood rather as a step towards new approaches to urban enquiry, more critically reflexive and sensitive to plural contexts (Robinson 2016).

At the same time, technology and the different infrastructures of the city are becoming increasingly complex and intertwined with both the governance of urban ecosystems and the shaping of the biophysical environment itself, with technology increasingly emerging as a third part in the human–nature relationship (Cronon 1991). Urban ecology is often hybrid, shaped by biophysical structures and processes inherent in ecosystems like shorelines, gardens, urban woodlands and rivers (green and blue infrastructure) and the built, human-engineered environment like buildings, sewers, roads and levees (e.g. Anderies 2014; Markolf et al. 2018). Ecological–technical designs are increasingly sought as instrumental solutions to various social and environmental issues (Depietri and McPhearson 2017; Maes and Jacobs 2017). For example, hybrid and novel ecosystems are wired and piped for automated watering, controlled by apps, or designed by engineers for water storage or other regulatory functions to complement grey infrastructure designs. The engineering approach stems from a different tradition than the ecological understanding and reconciling ideas about set and controllable standards, acceptable variance with emergence, cyclicity and non-human agency needs much more work.

In addition to the intersection of different infrastructures, hybridity also manifests in how we understand and manage urban social–ecological(–technological) systems. We see growing differences where many cities increasingly rely on information and communication technology technology for monitoring and informing management, while others choose different paths (Brondizio et al. 2016). The use of technology may exacerbate the urban human–nature disconnect, removing people either from the experiential learning much of the literature focus on, or from learning altogether, outsourcing learning and decisions to technology itself (Markolf et al. 2021). The implications of this increasingly digital approach to learning and relating to the system will likely have profound consequences (Galaz et al. 2021), although what they will be is less clear. Studies of human–nature relations share an appreciation of knowledge and learning as an essential cornerstone (e.g. Berkes and Folke 1998), and there is a very rich literature on the plurality of local ecological knowledge, traditional knowledge, indigenous knowledge, actionable knowledge, transformational knowledge and other knowledges. What happens when learning processes and sometimes knowledge holders are digital is a research frontier in need of more work.

This development leaves us with more ways of knowing the ‘system’ and, increasingly, with a need for different ways of connecting these understandings. Rather than trying to include all perspectives and all potentially relevant factors in a grand theory we point to a more pragmatic approach to growing more systemic understandings of the role and place of urban ecology. Whatever the scope and the starting point of an individual study, there is potential for expanding and making it contribute to a more systemic understanding. How this happens, across the diverse range of realities that file under the label ‘urban’, is a challenge that requires us to reflect on how knowledge is produced and used (see e.g. Muñoz-Erickson 2014). The next section gestures to useful ways forward though does not seek to provide “the answer” or prescribe best practices.

## Expanded contextualisation and methodological approaches

Systems framings and analyses can be used for many purposes, what we focus on here is the ability to position and relate individual phenomena or features to contextual circumstances. With expanding contextualisation—ontological, epistemological, and geographical—phenomena will start to overlap and connect. For example, the results of in-depth ecological surveys of the functional composition of urban species assemblages (understood as a contributing factor to different ecosystem services) can potentially be discussed in terms of the distributional justice of benefit sharing (e.g. Schell et al. 2020; Andersson et al. 2021a), or the implications of institutional arrangements of landownership and entitlements, or ecological literacy.

Methodologically, we need approaches that can be applied to very different situations *and* help us connect the results. This is made evident by issues of variable data availability, differences in resources, competences, and the general complexity of social–ecological–technological systems (SETS; Grimm et al. 2016; McPhearson et al. 2016a, b; McPhearson et al. 2022). In response, many scholars argue for improved epistemological agility (e.g. Haider et al. 2018), pragmatism (e.g. Moore 2016), and more integrated methods (Hitchcock and Onwuegbuzie 2022). Below we describe five complementary approaches for working with available evidence and building comprehensive knowledge that, individually or in combination, could help turn the conceptual challenge of assessing and evaluating equitable access to, and sustainable use of ecosystem-based wellbeing benefits, in recognition of the intrinsic values and rights of nature itself into a roadmap for future studies (Table 1).

**Table 1:** Five approaches for building systemic understandings

CATAR	Principles and use	Examples
**C**omparative approaches	Methods for qualitative and quantitative case comparison. Translation of findings across situations and cases, assessment of situatedness	Urban Comparative Studies (e.g. Robinson 2016), BIG DATA, modelling
**A**lternative approaches	Alternative ways of knowing and potentially substitutable methods. Accommodates differences in competence and context and provides opportunities for comparing methods	Human or automated field surveys, or citizen science (e.g. Stahl Olafsson et al. 2022)
**T**iered approaches	Data-driven, stepwise more sophisticated methods designed to work with coarse to complex, high-resolution data. Guidance on what kind of advice that can be based on different tiers	Ecosystem service assessments (Grêt-Regamy et al. 2015), different types of review protocols (from exploratory and scoping to systematic)
**A**ggregative approaches	Integrated and sequential methods for building a cumulative understanding. Core to building a systemic understanding and can draw on any and all of the above	Resilience and equity assessments (Andersson et al. 2021b; Grabowski et al. 2023), case study research (e.g. Feagan et al. 2019)
**R**eflexive approaches	Methods for examining how knowledge is produced and used, comparisons of how we learn about the system. Extended assessments of positionality and evaluation of implications	Project evaluations, monitoring of transdisciplinary learning processes (e.g. Mascarenhas et al. 2021)

### Comparative approaches

Learning from examples and other cases is the first of our approaches. It is common practice within and outside academia, but the complexity and situatedness described earlier in this article suggest comparison must be grounded in a broad and plural understanding of context. The transfer of knowledge from one case to another requires translation rather than replication. Similar to discussions elsewhere (e.g. on boundary objects (Star and Griesemer 1989) and concepts (Opdam et al. 2015)), one suggestion from comparative urban studies is to look for *comparators*, the features or concepts that may establish comparability of cases (Jacobs 2012). We see promise in the accumulation of global protocols and frameworks for assessing, evaluating and not least connecting new information. For example, expanding work around functional species traits position them as both a way for assessing the functional implications of different community assemblages and as a connector between biodiversity and people (e.g. de Bello et al. 2010; Andersson et al. 2021c; Grilo et al. 2022). Regional and global initiatives for compiling and cataloguing traits information (e.g. TRY Kattge et al. 2011 and CESTES Wolff et al. 2019) offer a basis for comparing ecological functionality, vulnerabilities and response diversity across regions and biomes (e.g. Lavorel and Garnier 2002; Hevia et al. 2017). Furthermore, the rapid expansion of new technology for sensing, sharing, and processing different types of information provides new opportunities for large scale comparison and benchmarking. At the same time the same progress highlights differences in access to technology and data (McHale et al. 2013; Rega-Brodsky et al. 2022), and global comparisons are often frustrated by different standards and protocols for how data are produced, measured, and interpreted (Creutzig et al. 2019).

With the shift from ecology towards social–ecological and even social–ecological–technological systems, there is now also a wide array of more qualitative methods potentially relevant for understanding the nature of cities (see e.g. Robinson 2016). Urban comparative studies has researched approaches that allow deeper understanding of urban systems by seeking to capture and discuss differences and similarities between two or more cases in order to contribute to an inductive exploration and theorisation (Collier 1993; Wolff and Haase 2020). Comparability can be sought in the commonalities of how a phenomenon is manifested across cities and situations, under the assumption that it follows the same rules (Robinson 2011) or in differences and contrast. Here, Robinson (2016) writes “what might be considered a ‘case’ needs to be redefined to avoid the restricting and territorialising trap of only comparing (relatively similar) ‘cities’: we might rather compare, for example, specific elements or processes in cities, or the circulations and connections which shape cities, thus, rendering urban experiences comparable across a much wider range of contexts, and building research strategies which are adequate to the complex spatiality of urban forms” (ibid, p. 5). Examples of specific elements include yards or gardens, forest patch configuration, networks of connectivity, or social economic heterogeneity.

Comparison allows investigation into situatedness and the role contextual factors may play for the emergence of different phenomena. It can also point to alternative explanations or additional factors that could be of relevance to the case. Geographic comparison can also connect a case to the global discussion of what urban means and how urbanisation unfolds differently in different parts of the world. The more inductive approach in urban comparative studies—seeing cases as loose assemblages of elements—also lends itself well to repositioning and re-evaluating the relation between different features, which is core to reflexivity (see below).

### Alternative approaches

With a growing interest in inter- and transdisciplinary approaches not least in sustainability science, it is becoming clear that there are many ways and methodological options for answering, or at least speaking to, the same or similar questions (e.g. Biggs et al. 2021). These designs draw on different data and involve different competences and ways of knowing (Tengö et al. 2017), and different modes of research (e.g. Lang and Wiek 2021). Heterogeneity and diversity within and across cities offer opportunities for natural experiments and observational research as well as action research. Researchers can either stand to the side or be embedded in and actively part of shaping the processes they study and the researchers apart from phenomena they study, with suites of methods to support either. The latter covers a range of more or less active roles from setting up ecological experiments that involve or in some other way engage the general public to actively setting up and facilitating transformation processes (e.g. Lang et al. 2012).

Different methods and approaches may also be more or less culturally appropriate (e.g. Wright et al. 2016), or offer a particularly good fit with a certain type of existing data. To study, for example, the human experience of nature one may examine the concepts and theories that are embedded in the situation and described in the scientific literature, or examine records of the experience, ranging from art and artefacts to official records or diaries, or interview and ask people directly (with or without secondary elicitation tools like photographs), or use fiction or poetry to explore the emotional tone (e.g. Roulston 2010; Kara 2015).

While more or less supplementary in terms of the knowledge they produce, alternative approaches may come with method specific added values. For example, data collection as well as analysis can be researcher or expert-led, automated, or co-created. Citizen or resident science can mobilise extensive and dispersed networks of volunteers to assist in research and provide a gateway for faster uptake of knowledge and/or the co-production of knowledge (e.g. Muñoz-Erickson 2014; Borgström et al. 2021). Citizen science has both the ability to generate observations at scales or resolutions unattainable by individual researchers (e.g. Cooper et al. 2007; Dickinson et al. 2012), and reach sites like private lands and residential areas otherwise not accessible to researchers (e.g. Kobori and Primack 2003).

The real contribution from supplementary methods to a more systemic understanding, however, comes from the application of several alternative methods in parallel. The increasingly diverse ways of asking questions will require more work on investigating and critically examining the type of answers you get through different ways of asking questions, and what the answers can be used for (see e.g. Johnston et al. 2020). Additionally, alternative methods may be more less easy to build on in aggregative approaches (see below). For example, choosing a citizen science design rather than automated design means that there will (or could be) metadata on the rapporteurs, which can be a first step towards understanding aspects of how people relate to e.g. biodiversity or climate change.

### Tiered approaches

If the alternative approach builds on supplementarity and making each study design unique and adapted to a specific situation, differences in the quality of available data and the availability of different tools can also be navigated with tiered approaches. Looking at ecosystem service mapping, Grêt-Regamey et al. (2015) suggested a tiered assessment system (building on Crossman et al. 2013), where the methods are decided both by available data and resources and the answers needed. The tiered system is interesting for a discussion about the type of answers different approaches provide, and what kind of advice that can be based on them. There is often an information cost threshold where more resources and finer detail no longer make a big difference for the answer you may reveal (e.g. Hayek 2011). Understanding types of answers and how they may fit into the uptake and application of science-based knowledge is essential for producing actionable knowledge. This captures another aspect of ‘systemic’ and the aspiration of urban ecology to become a more integral, actionable part of urban development.

Rapid but patchy developments in the field of remote sensing is one place where a tiered approach could be a good fit. For example, while advances in use of high-resolution hyperspectral data, light detection and ranging (LiDAR) or mounting sensors on unmanned aerial vehicles (UAVs) or embedded in urban infrastructure provide new tools that tools that can expand the range of potential measurements, older technology still supports thematically similar if coarser analyses. Another example would be where the first tier uses only a single method, which in higher tiers is complemented with additional methods, for example adding a qualitative understanding to a quantitative base. This approach combines the tiered approach with the aggregative approach below.

More generally, existing tiered frameworks like Ostrom’s (2007) systematic diagnosis offer positioning tools and guidance for how to think about at what conceptual level individual research items are located, the evidence more readily available and decide on a manageable combination of variables to be analysed.

### Aggregative approaches

Over time, narrowly framed studies can be turned into more comprehensive cases, and a rich, deep case understanding makes positioning and connecting new information easier. This expansion can be done by connecting a new study to other studies focusing on other, ‘adjacent’, fields within the conceptual framework (see e.g. Branny et al. 2022), in our case the sustainable production and use of ecosystem-based wellbeing benefits. For example, ecosystem service assessments can focus on the values and perspectives held by users, the practices of service mobilisation and realisation or the ecological basis for potential services. Using this systemic relationship, narrow, bounded studies can connect and jointly provide a richer understanding that can support further innovation in research and practice. It can also make commonalities between cases easier to find (see e.g. Ziegler 2013), which can then be used for comparison.

Building a ‘new’ case for every study is a substantial task and often not doable within tight time frames or with limited resources, thus, underlining the importance of long-term studies in urban ecology. What methodological development could do, not least on integrated mixed- or multi-methods approaches (see e.g. Hitchcock and Onwuegbuzie 2022), is to make the gradual accumulation of comprehensive cases easier. With more options for bricolage and directly building on what is already there, cases could be assembled more quickly and individual studies more broadly grounded. Alternatively, patterns observed through monitoring, for example through citizen-science-acquired data, may elicit questions that spark new studies with more sophisticated or far-reaching researching research questions (e.g. not only what is the trend but why do we see it).

Sequential study designs lend themselves well to addressing questions that arise and go beyond the scope of a first study, or to move a project or new knowledge toward implementation (e.g. Tengö et al. 2017; Andersson et al. 2021a, b, c), for example by connecting, adapting and applying general knowledge to case specific policy guidelines and strategies (e.g. Borgström et al. 2021). The later steps in a sequential design tend to draw on supplementary data and use different methods. The methods, or the interpretation of their results, are informed by and to some extent dependent on the previous steps, i.e. they may use or add data that are not saturated or fully match the research questions (e.g. Hesse-Biber and Johnson 2015; Maxwell 2016). The sequence then continues until the researcher is certain enough that their analysis regarding the full set of research questions (or, as it were, a larger part of the systems conceptualisation has been addressed) are answered.

### Reflexive approaches

Expanding a systems understanding also means going beyond a descriptive-analytical approach to the system and its phenomena as ‘objects’. We suggest a pragmatist’s approach (e.g. Cherryholmes 1992; Putnam 2002; Bhaskar 2009; Turnheim et al. 2015) to knowledge production, in the sense that we challenge the reductionist model of objective positivism and the presumed dichotomy between production and use of knowledge. We see growing and constantly changing understandings of the system as an opportunity for reflexivity, and reflexivity as a means for enriching what is understood to be the system. Any of the approaches above, and hybrids combining several of them, provide learning opportunities in themselves, and opportunities for comparing processes and practices related to exploration and knowledge generation. In our understanding of a social-ecological-technological system, self-awareness and the continuous deliberative and learning practices of people engaged in joint exploration, sense-making and enacting are part of the system itself and not a parallel process of studying it.

As cases become entangled in their contexts and interpretations (i.e. implications for social, environmental or more-than-human justice), cases can be deliberately and inferentially connected by the application of the same analysis–valuation–evaluation frameworks, or a comparison of how the cases were derived. Following Popa et al. (2015), we see reflexivity as a practice of “(1) deliberation on the overall normative and epistemic orientation of the research; (2) deliberation on the socially relevant framing of research problems; (3) generation of reflexivity on values and understandings in concrete problem-solving and social experimentation processes; (4) generation of reflexivity on normative commitments and ideological orientations in social transformation processes” (ibid, p. 50). The increasingly methodologically and theoretically rich field of urban enquiry offers grounds for not just building urban systems knowledge, but to make this a living knowledge system, situated in academic and non-academic values and institutional arrangements and power structures.

Constantly revisiting assumptions and looking for alternative explanations will strengthen the other approaches. Reflecting and being more transparent about positionality (normative and epistemic as well as in relation to the study subject) provides another layer of understanding and potential inquiry.

### Intersections and convergence

What, then, is the systemic principles to bind them all? Individually or combined, the approaches benefit from a framework that helps you situate different bits of knowledge. Positioning individual studies and methods within a system context allows for different levels of contextualisation and extended possibilities for interpreting implications and connecting to other cases or studies. Still, building cohesive, comprehensive, and systemic knowledge poses its own challenges in terms of finding common frames and ground for comparison and complementarity. Comparison and generalisability have often sought quantitatively, at least within the natural sciences (e.g. Wu and Loucks 1995). However, as positioned in this paper, a systemic approach needs to accommodate also other traditions interested in the different scales and factors shaping human–biodiversity relations. Comparison and aggregation build on some of the same premises. The basic question is what is it that cases (and cases will be diverse and heterogeneous) share that can make them comparable? Building on, amongst others, Kronenberg and Andersson’s (2019) work on integrated valuation, we propose a first relatively simple framework for thinking about comparability and complementarity is to pay attention to and work with the *compatibility* and *commensurability* of the theoretical frameworks, conceptual orientation, and methods and their underlying data, i.e. their ability to fit together and contextualise each other.

We propose using compatibility to capture the more practical, mechanistic and technical aspects of different studies, i.e. the processes and tools used for data generation and analysis, the types of data produced or used, potential explanatory factors and causal mechanisms, types of units analysed, and scale(-s) used. Commensurability instead refers to the underlying research perspective of the study and the set of methods it includes, i.e. the ontological and normative assumptions it builds on and the, sometimes subjective, frames that are used to evaluate outcomes. It also captures the position within conceptual space, and the degree of contextualisation. Work is still needed to turn the two terms fully operational, but as guidelines and integrators for interdisciplinary and systems-oriented studies we hope this development may serve as a conduit for scientific advancement of urban ecological understanding embedded in complex world cities.

### Building comprehensive understanding(s)

While the five approaches can be used in many ways to support more systemic understandings, we here provide one hypothetical example for guidance or inspiration. The systemic perspective we advocate includes both what you study and how you study it. These two axes and the assessment of commensurability and compatibility can serve as a first positioning of a study or knowledge item based on research questions and the methods used. Earlier in the article we put the sustainable production and use of ecosystem-based wellbeing benefits as a centre piece in ‘the urban system’, and this will allow you to place studies along a continuum ranging from ecosystem dynamics and generation of ecosystem services, via the mobilisation and appropriation of services, to the values we attribute to them. This conceptualisation opens up additional questions of how a targeted, narrow study relates to the other aspects (e.g. how and by whom are potential services used and valued?). Initially, without information on the intermediary steps, studies on ecosystem functions and value attribution may seem rather incommensurable, but the conceptual/theoretical framing and additional information on the space between them, in this case the mobilisation and realisation of ecosystem services, can make them easier to connect (see e.g. Meacham et al. 2022). Similarly, methods and metrics provide the study with another set of coordinates and another potential comparator. Expanding the scope of a study and adding research questions means adding data or new analyses, and a tiered diagnosis existing data can provide an indication of the type of outcomes you can expect and what you can use them for (like e.g. Grêt-Regamey et al. 2015).

Asking more questions will, usually, also mean using additional methods (to provide missing data or provide new analytical tools). These may dig deeper into the case employing sequential and integrated methods or allow you to triangulate by comparing alternative ways of interrogating your case. Another option is to compare cases and ask under which circumstances for example the ecosystem service cascade is realised in certain ways (the actors and practices involved, the institutions framing resource use). In either approach, the frameworks used for evaluating and discussing outcomes offer a way to compare outcomes that also lends itself to discussions about the implications and desirability of different outcomes. Connecting to, for example, the discourses on justice or resilience, offer yet another set of coordinates that will help relate and position a study relative to others and ongoing discussions about more or less desirable pathways for future urban development. Finally, reflexive practice is becoming increasingly used in interdisciplinary efforts, and this may serve as an extra intermediary or bridge between more ‘distant’ studies. For example, as studies of disaster and consideration of inequitable vulnerability in the face of extreme events, reflexivity in both realms has helped identify legacies of prior disturbance or localised disadvantage as a bridge concept. Beyond the methods used there is the actual practice and experience of doing research (e.g. being an observer or an active participant on the case you study are two very different positions), and these too can be connected, shared and compared.

## Conclusions

What would it take to better position, relate, and connect different studies and build bridges between different lines of research? First, richer case descriptions and use of one or more of the above denominators (or other broad, unifying themes like justice or wellbeing) would make it easier to move studies from parallel (at best) to combination. Second, as we have indicated there is much to be done in terms of improving methodological compatibility and our ability to compare, contrast and evaluate outcomes and insights produced differently. While the above ‘convergence’ approach is pragmatic and perhaps somewhat positivistic, we do not want to downplay epistemological differences between different traditions. Synthesis is possible, doable but not easy. What we propose is more effort dedicated to better understanding and communicating what different approaches and tiers of knowledge afford.

While not inherently a requirement for applied urban ecology or the study of social–ecological–technological systems, many studies are intended to be policy relevant, sometimes even policy informing. For this to be realistic and the suggestions credible there is yet another layer of study that needs to be added to the urban case. Even with more comprehensive cases and better methods for translating insights across cases our understanding and the type of advice science can offer will vary. Still, incomplete understanding can also be useful as long as the limitations are clear, especially given the perceived urgency of many social–environmental problems begging for input now, not later when we perhaps know better.
